# AZA-MS: a novel multiparameter mass spectrometry method to determine the intracellular dynamics of azacitidine therapy *in vivo*

**DOI:** 10.1038/leu.2017.340

**Published:** 2018-01-16

**Authors:** A Unnikrishnan, A N Q Vo, R Pickford, M J Raftery, A C Nunez, A Verma, L B Hesson, J E Pimanda

**Affiliations:** 1 Adult Cancer Program, Lowy Cancer Research Centre, UNSW Sydney, Sydney, New South Wales, Australia; 2 Prince of Wales Clinical School, UNSW Sydney, Sydney, New South Wales, Australia; 3 Bioanalytical Mass Spectrometry Facility, UNSW Sydney, Sydney, New South Wales, Australia; 4 Climate Change Cluster, University of Technology Sydney, Sydney, New South Wales, Australia; 5 Department of Pathology, School of Medical Sciences, UNSW Sydney, Sydney, New South Wales, Australia; 6 Haematology Department, Prince of Wales Hospital, Randwick, New South Wales, Australia

## Abstract

The cytidine analogue, 5-azacytidine (AZA; 5-AZA-cR), is the primary treatment for myelodysplastic syndrome and chronic myelomonocytic leukaemia. However, only ~50% of treated patients will respond to AZA and the drivers of AZA resistance *in vivo* are poorly understood. To better understand the intracellular dynamics of AZA upon therapy and decipher the molecular basis for AZA resistance, we have developed a novel, multiparameter, quantitative mass spectrometry method (AZA-MS). Using AZA-MS, we have accurately quantified the abundance of the ribonucleoside (5-AZA-cR) and deoxyribonucleoside (5-AZA-CdR) forms of AZA in RNA, DNA and the cytoplasm within the same sample using nanogram quantities of input material. We report that although AZA induces DNA demethylation in a dose-dependent manner, it has no corresponding effect on RNA methylation. By applying AZA-MS to primary bone marrow samples from patients undergoing AZA therapy, we have identified that responders accumulate more 5-AZA-CdR in their DNA compared with nonresponders. AZA resistance was not a result of impaired AZA metabolism or intracellular accumulation. Furthermore, AZA-MS has helped to uncover different modes of AZA resistance. Whereas some nonresponders fail to incorporate sufficient 5-AZA-CdR into DNA, others incorporate 5-AZA-CdR and effect DNA demethylation like AZA responders, but show no clinical benefit.

## Introduction

The cytidine analogue, 5-azacytidine (AZA; 5-AZA-cR), is the primary disease modifying pharmacological agent currently available for the treatment of the haematological neoplasms, myelodysplastic syndrome (MDS) and chronic myelomonocytic leukaemia (CMML). The efficacy of AZA compared with supportive care alone has been shown in MDS^1^ and CMML.^2, 3^ However, only approximately half of AZA-treated MDS or CMML patients respond to treatment and response is associated with improved survival outcomes and decreased likelihood of leukaemic transformation.^1, 4^ Although some clinical parameters^3, 5^ and genetic mutations^6, 7^ have weak correlations with favourable AZA response, the molecular mechanisms underlying primary AZA resistance are poorly understood. Furthermore, AZA response is rarely sustained and a significant fraction of patients who initially respond will eventually relapse within a 2-year period, with very poor subsequent prognosis thereupon.^8^

Following cellular uptake, AZA is metabolised and incorporated into DNA and RNA.^9^ AZA triphosphate, in its deoxyribonucleic form 5-aza-2′-deoxycytidine (DAC, 5-AZA-CdR) triphosphate, mediates DNA demethylation via the covalent trapping of DNA methyltransferases^10^ that eventually leads to the degradation of the methyltransferases through a proteasomal mechanism.^11^ However, it is unclear whether AZA-induced DNA demethylation is responsible for its efficacy in MDS and CMML. Although AZA therapy certainly causes DNA hypomethylation in patients,^12^ hypomethylation is not predictive of clinical response.^13^ In addition, studies have failed to find any correlation between AZA-mediated DNA demethylation and subsequent gene re-expression,^14, 15^ suggesting additional molecular mechanisms might be at play *in vivo* to explain the efficacy of AZA. Furthermore, recent data have implicated immune response because of double-stranded RNA production from endogenous retroviral elements as one mode of AZA efficacy.^16, 17^ Some of the mechanisms proposed for primary AZA resistance have included insufficient intracellular concentration of AZA triphosphates, either because of insufficient intake of AZA through membrane transporters, deoxycytidine kinase deficiency, excessive deamination by cytidine deaminase or high intracellular nucleotide pools^18^ that could all potentially limit 5-AZA-CdR levels in DNA. Alternatively, increased cell cycle quiescence of haematopoietic cells, limiting the DNA replication-dependent incorporation of 5-AZA-CdR triphosphates, has also been proposed as a marker of AZA resistance.^19, 20^ Although most studies have focused on the DNA demethylation effect resulting from DNA incorporation of 5-AZA-CdR triphosphates, metabolic labelling studies have suggested that the majority of AZA (80–90%) is in fact incorporated into RNA as AZA triphosphates.^21^ Furthermore, comparative studies of AZA and DAC (native 5-AZA-CdR) therapy have shown that they display different effects on cell viability and gene expression,^22^ suggesting that AZA incorporation into RNA might have distinct consequences. AZA incorporation into RNA leads to the covalent trapping of RNA methyltransferases and demethylation of some RNAs,^23, 24^ as well as the destabilisation of other transcripts.^25^ However, the overall biological consequences of AZA incorporation into RNA are poorly understood, as is any potential role in mediating therapeutic response.

The development of analytical methods to measure AZA dynamics *in vivo* has been constrained by the marked chemical instability of AZA in aqueous solutions^26, 27, 28, 29^ that can greatly decrease the abundance of intracellular AZA and require technologies with very high detection sensitivity. Additionally, the small molecular weight difference (1 da) between AZA or 5-AZA-CdR and endogenous cytidine or deoxycytidine (dC) respectively requires technologies with high resolution to accurately quantify AZA or 5-AZA-CdR levels intracellularly. Although radiolabelled AZA followed by scintillation-based quantification of subcellular components has been applied to samples *ex vivo*,^30^ this technology is not readily amenable to studying intracellular AZA dynamics *in vivo*. Mass spectrometry (MS), with its high resolution and sensitivity, has been applied to study AZA or 5-AZA-CdR triphosphate levels in samples from patients undergoing treatment,^25, 31, 32, 33^ but these methods have been unable to assess AZA or 5-AZA-CdR levels into DNA or RNA simultaneously within the same samples, limiting an overall picture of AZA intracellular dynamics from being painted. For these reasons, we have developed a novel liquid chromatography (LC)–MS method (termed ‘AZA-MS’) capable of quantifying multiple parameters in parallel within the same fraction of AZA-treated cells.

## Materials and methods

### Cell culture and treatment

RKO cell line (ATCC, Manassas, VA, USA) was cultured in RPMI-1640 medium containing 10% fetal bovine serum, 100 U/ml penicillin, 50 U/ml streptomycin and 2 mM GlutaMAX (Thermo Fisher Scientific, San Jose, CA, USA) and was maintained at 37 °C in a humidified environment with 5% CO_2_. Cells were treated with different concentrations of 5-azacytidine (kind gift, Celgene, Summit, NJ, USA) for 72 h, with media change containing fresh drug every 24 h.

### Primary bone marrow samples from MDS and CMML patients

Bone marrow samples used in this study were obtained from patients recruited from New South Wales, Australia, on a compassionate access basis for AZA monotherapy. All samples were obtained with written informed consent in accordance with the Declaration of Helsinki and approval of the South Eastern Sydney Local Health District human research ethics committee. WHO (World Health Organisation) classification,^34^ IPSS-R (International Prognostic Scoring System - Revised)^35^ and CPSS (CMML-Specific Prognostic Scoring System)^36^ scoring and AZA response^37^ were based on published guidelines. AZA treatment (75 mg/m^2^) consisted of standard 28-day cycles, and bone marrow samples from each patient were collected before treatment, at the end of the first 7 consecutive days of subcutaneous AZA (C1d8) and following 21 days of intermission at the end of cycle 1 (C1d28). Immediately upon sample collection, mononuclear cells were isolated from the bone marrows by density centrifugation using Lymphoprep (Stem Cell Technologies, Vancouver, BC, Canada). Mononuclear cells were incubated with CD34 magnetic beads (Miltenyi Biotec, Bergisch-Gladbach, Germany) and separated using an AutoMACS Pro machine (Miltenyi Biotec) exactly as per the manufacturer’s recommendations. Viably frozen vials of CD34− cells from the relevant time points were thawed, washed once in phosphate-buffered saline and used to prepare RNA, DNA and cytoplasmic extracts for AZA-MS (as outlined below).

### AZA-MS method

#### DNA/RNA extraction

All samples were washed with phosphate-buffered saline solution containing 100 μg/ml tetrahydrouridine (Abcam, Cambridge, UK) and frozen at −80 °C. DNA and RNA were purified from frozen cell pellets using the All-In-One DNA/RNA Miniprep Kit (Astral Scientific, Sydney, NSW, Australia) following standard manufacturer recommendations, including RNase and DNaseI treatments to remove contaminating RNA or DNA as appropriate. Nucleic acids were quantified using the NanoDrop ND-1000 Spectophotometer (NanoDrop Technologies Inc., Thermo Fisher Scientific). Purified DNA and RNA samples were stored at −80 °C until further processing.

#### DNA/RNA preparation for LC–MS analysis

For duplicate measurement, 1–10 μg of extracted DNA, together with 5 μl of 50 μM 5-aza-2′-deoxycytidine-^15^N_4_ (internal standard, Toronto Research Chemicals, Toronto, ON, Canada), was added to 10 μl of 20 mg/ml NaBH_4_ solution. In case of RNA, 1–10 μg of extracted RNA, together with 5 μl of 50 μM 5-azacytidine-^15^N_4_ (internal standard, Thermo Fisher Scientific), was added to 10 μl of 20 mg/ml NaBH_4_ solution. The mixture was incubated at room temperature with agitation for 20 min, and neutralised with 1 μl 5 M HCl. Reduced DNA or RNA were digested with 1 h of incubation at 37 °C following the addition of 40 μl of Digest Mix, an aqueous solution containing 50 U/ml benzonase (Sigma Aldrich, St Louis, MO, USA), 60 mU/ml phosphodiesterase (Sigma Aldrich), 20 U/ml alkaline phosphatase (Sigma Aldrich), 20 mM Tris HCl pH 8, 100 mM NaCl and 20 mM MgCl_2_ as previously described.^38^ Samples were dried under vacuum (Savant Speedvac Plus SC210A, Thermo Fisher Scientific) and resuspended in 50 μl of CE buffer (10 mM Tris HCl pH 8.0 and 0.5 mM EDTA in Milli-Q water, Merck Millipore, Bedford, MA, USA) for LC–MS analysis.

#### Cytoplasmic extract preparation for LC–MS analysis

Frozen cell pellets were resuspended in phosphate-buffered saline containing 100 μg/ml tetrahydrouridine. Cells were lysed by thorough mixing with absolute MeOH, followed by addition of internal standards. Samples were centrifuged at 12 000 r.p.m. for 10 min at 4 °C and the supernatants were dried under a vacuum. The resulting precipitates were resuspended in CE buffer and the mixture was reduced, neutralised and digested with an enzymatic mix as described earlier for DNA and RNA preparations. Samples were then dried under vacuum, resuspended in 50 μl of CE buffer and utilised for LC–MS analysis.

### Mass spectrometry and data analysis

LC–MS analysis was performed utilising an ultra-high-performance liquid chromatography system (Dionex u3000 system, Thermo Fisher Scientific) interfaced to an Orbitrap mass spectrometer (Q Exactive Plus, Thermo Fisher Scientific) using a heated electrospray interface operated in the positive ion mode. The chromatographic separation was performed on a 100 mm × 2.1 mm i.d., 3 μM, C30 column (Acclaim, Thermo Fisher Scientific) kept at 40 ºC. Then, 20 μl of the sample was analysed using gradient elution with 0.1% formic acid in Milli-Q water (Solvent A) and 0.1% formic acid in acetonitrile (Solvent B) at a flow of 0.4 ml/min over 8 min (Table 1).

Mass spectra were acquired at a resolution of 140 000 over the range of 220 to 260 Th. The electrospray voltage was set to 4000 V. The sheath gas pressure and the auxillary gas pressure were 5 and 5 (ThermoFisher arbitrary units), respectively. The capillary temperature was 300 °C and the s-lens was 80 V. Data processing of chromatograms was performed using the Quanbrowser function of the Xcalibur Software package version 2.5 (Thermo Fisher Scientific). Quantification was performed on analyte-specific peaks obtained using accurate mass extracted ion chromatograms (Table 2).

Calibration standard mixes containing all analytes (AZA, 5-AZA-CdR, C, dC, mC and mdC) were prepared by dilution in CE buffer of the following chemicals: 5-azacytidine and decitabine (Selleckchem, Houston, TX, USA), cytidine triphosphate and deoxycytidine triphosphate (Promega, Madison, WI, USA) and 5-methylcytidine and 5-methyl-2′-deoxycytidine (MP Biomedicals, Irvine, CA, USA). To 5 μl of each standard mix were added 5 μl of 50 μM 5-aza-2′-deoxycytidine-^15^N_4_ and 5 μl of 50 μM 5-azacytidine-^15^N_4_ internal standards. The mixture was reduced, neutralised and digested as described earlier. Samples were dried under vacuum and resuspended in 50 μl of CE buffer, bringing the final concentration ranges to: AZA and 5-AZA-CdR (0.01−1 μM), C and dC (1−50 μM) and mC and mdC (0.02−1 μM). Standard curves were created by plotting the ratio of the peak area of the analyte to the internal standard ratio, against analyte concentration, using a least-squares polynomial regression. The standard curves were then used to quantify the analyte within the quality control (QC) and test samples. To obtain the quantity of incorporated AZA or 5-AZA-CdR per unit of DNA or RNA, the following equation was used to convert the readout from the standard curve: ([Interpolated value (pmol/μl) × injection volume (μl))/DNA input (μg). Global RNA and DNA methylation levels were assessed by calculating the ratio of mC to total C (mC+C) or mdC to total dC (mdC+dC), as previously reported.^39^ Correlation was measured by calculating Pearson’s product-moment coefficient. All statistical analyses were performed in GraphPad Prism (GraphPad Software Inc, San Diego, CA USA).

### Quality control assessments

QC samples were prepared by spiking in defined quantities of DAC into a fixed amount of genomic DNA from untreated RKO cells, or AZA into genomic RNA from untreated cells, at high (1 μM), medium (0.3 μM) or low (0.03 μM) levels. QC samples were analysed in duplicate in each run. The sensitivity was determined from the signal-to-noise ratio in the lower limit of quantitation sample in three separate experiments. Specificity was established by examining for any interfering peaks within the range of the specific analyte being measured, in untreated RKO samples (for AZA or 5-AZA-CdR) or within blank digest matrix (for C, mC, dC and mdC). Precision and accuracy analyses were calculated from replicate data acquired on three separate days. Eight point standards and QC samples (representing low, medium and high quantities of the analyte) were included in duplicate in each run.

### Gene expression measurements

Gene expression levels of the cell cycle genes were determined by quantitative real-time PCR as previously reported.^19^ RNA was extracted from primary pretreatment bone marrow CD34+ cells using the All-In-One DNA/RNA Miniprep Kit (Astral Scientific) following standard manufacturer recommendations. Complementary DNA was subsequently prepared from RNA using the QuantiTect Reverse Transcription Kit (Qiagen, Hilden, Germany) as per the manufacturer’s recommendations. Quantitative real-time PCR was performed on a Stratagene M × 3000P machine (Agilent, Santa Clara, CA, USA) using Express SYBR GreenER qPCR Supermix Universal and ROX reference dye (Thermo Fisher Scientific). Relative expression levels were calculated from Ct values and three reference genes as per established guidelines.^40^

## Results

### Establishing a high-resolution mass spectrometry method to directly quantify intracellular 5-AZA-CdR

**Figure 1:**
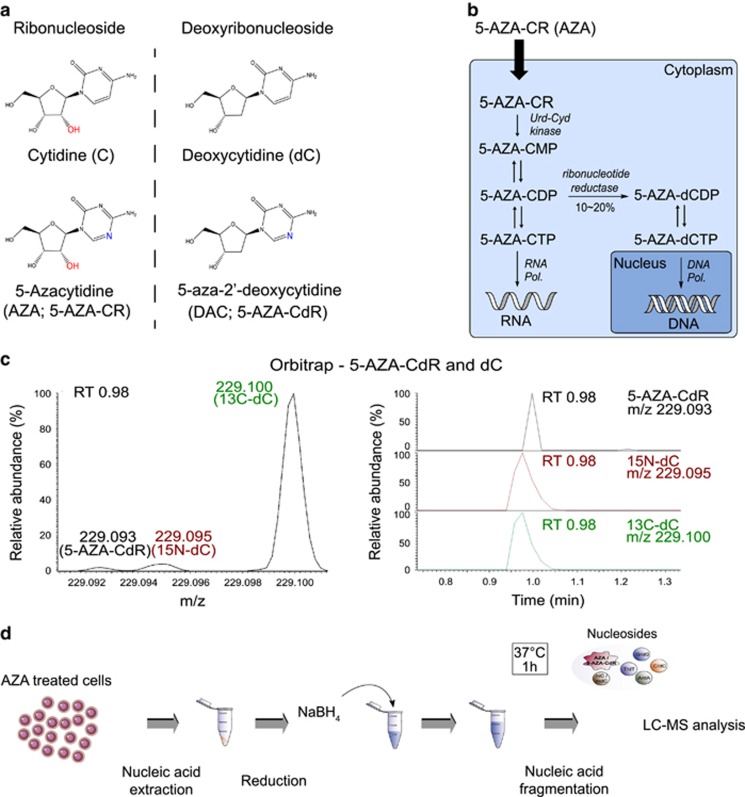
Development of a quantitative high-resolution mass spectrometry to directly measure intracellular 5-AZA-CdR and AZA. (**a**) Chemical structures of cytidine, deoxycytidine, AZA and 5-AZA-CdR. (**b**) Schematic depicting intracellular metabolism of AZA. Following cellular uptake and phosphorylation, ~80% of AZA gets incorporated into RNA by RNA polymerases. The remaining fraction is converted into 5-AZA-CdR by ribonucleotide reductase and incorporated into DNA by DNA polymerases. (**c**) Representative high-resolution Orbitrap mass spectra at RT of 0.98 min, showing clear baseline separation between 5-AZA-CdR, 15N-dC and 13C-dC (left, with respective *m/z* values) despite their identical chromatographic retention times (right). (**d**) Schematic depicting the optimised method incorporating steps to improve sensitivity of detection of intracellular 5-AZA-CdR and AZA by LC–MS.

5-Azacytidine (AZA; 5-AZA-CR) is an analogue of the ribonucleoside, cytidine, whereas 5-aza-2′-deoxycidine (DAC; 5-AZA-CdR) is an analogue of the deoxyribonucleoside, deoxycytidine (Figure 1a). Following AZA therapy and cellular uptake, AZA is phosphorylated intracellularly into the triphosphorylated ribonucleotide (5-AZA-CTP, Figure 1b) and then incorporated into RNA. A small fraction of intracellular 5-AZA-CDP is converted into a deoxyribonucleotide, 5-AZA-CdR diphosphate (5-AZA-dCDP, Figure 1b) by ribonucleotide reductase. After a further phosphorylation step, 5-AZA-dCTP can be incorporated into DNA, where it can affect DNA demethylation through the covalent trapping and proteasomal degradation of DNA methyltransferases. In this manner, therapeutically administered AZA can end up in both RNA and DNA intracellularly. As the focus of this study is the measurement of synthetic and native nucleosides in DNA and RNA following AZA therapy, we will use the term 5-AZA-CdR for the AZA-derived deoxyribonucleoside instead of DAC to avoid confusion with its therapeutic form.

We first sought to develop a quantitative LC–MS-based method to accurately measure the amount of 5-AZA-CdR within cells without interference from deoxycytidine (dC), a mass difference of just 1 Da. As a first step, we evaluated the applicability of a previously established LC–MS method for quantifying dC and 5-methyl-2′-deoxycytidine levels in DNA^41^ using a high-sensitivity triple quadrupole mass spectrometer. Comparing a sample containing only dC to another containing only 5-AZA-CdR, we observed an interference signal in the *m/z* window corresponding to 5-AZA-CdR in the dC-only sample (Supplementary Figure 1a). We established that the interference was arising from the ^15^N- and ^13^C-containing natural isotopes of dC (Supplementary Figure 1b). Given the very close *m/z* values for 5-AZA-CdR (229.093), ^15^N-dC (229.095) and ^13^C-dC (229.101), we reasoned that a new-generation Orbitrap mass spectrometer (Q Exactive Plus, ThermoFisher) with a mass resolution of 280 000 (full width at half maximum) would have more than sufficient capability to separate out 5-AZA-CdR from the different isotopes of dC. Using spiked test samples as before, we achieved direct mass resolution of 5-AZA-CdR from ^15^N-dC and ^13^C-dC on the Orbitrap (Figure 1c).

### Quantifying DNA-incorporated 5-AZA-CdR intracellularly with high sensitivity

Intracellularly, nucleosides can exist as mono-, di- and tri-phosphorylated nucleotides. In order to reduce the analytical complexity associated with measuring the various isoforms of all of the different nucleotides, we adopted a nucleic acid fragmentation method that yields dephosphorylated nucleosides.^38^ A major further complication is the marked chemical instability of AZA and 5-AZA-CdR in aqueous environments,^26, 27, 28, 29^ with reported half-life of as low as 7 h at 37 °C. As the original DNA fragmentation method required 6 h of incubation step at 37 °C, we tested whether the prolonged incubation at this temperature would be detrimental to 5-AZA-CdR (and by corollary, AZA). Indeed, we observed a more than twofold decrease in detectable signal by MS consistently across a range of 5-AZA-CdR concentrations tested, following 6 h of incubation at 37 °C (Supplementary Figure 2a).

We sought to mitigate the hydrolysis problem and thereby improve the sensitivity of detection by a two-pronged approach. First, we established the minimum amount of time required to completely fragment DNA. Testing a range of fragmentation times (1, 2 or 6 h) and different input DNA concentrations (1, 2 and 5 μg), we determined that 1 h was sufficient to fragment up to 5 μg of DNA (Supplementary Figure 2b). Second, we aimed to decrease the rate of spontaneous hydrolysis of AZA or 5-AZA-CdR in aqueous solutions. A major mode of decomposition is through hydrolytic ring opening of the labile 5,6-bond of the triazine ring followed by deformylation.^27, 29, 42^ We examined whether the reduction of this bond with sodium borohydride, yielding dihydro-5-AZA-CdR, would improve the detection sensitivity. Indeed, across a range of 5-AZA-CdR concentrations tested (0.5−1000 nM), we observed a greater than twofold improvement in signal for 5-AZA-CdR (in its reduced dihydro-5-AZA-CdR form, Supplementary Figure 2c). A similar improvement in signal upon reduction with sodium borohydride has also been previously reported for AZA.^25^ We also validated that our reaction conditions resulted in complete reduction of all AZA and 5-AZA-CdR present (Supplementary Figures 2d and e). For the sake of simplicity, all future instances of 5-AZA-CdR or AZA are implied to mean measurements of dihydro-5-AZA-CdR or dihydro-AZA respectively.

To further improve sensitivity of detection, we set about systematically evaluating a range of different parameters to identify attributes that might further improve the sensitivity of detection. We ascertained that removing ammonium formate from the chromatographic buffer resulted in a 27-fold improvement in signal intensity for 5-AZA-CdR across a range of 5-AZA-CdR concentrations tested (Supplementary Figure 2f). Evaluating a range of chromatographic columns, including a C18 HSS, C18 BEH and a C30 column, we identified the C30 column as the most optimal (data not shown). We also optimised the *m/z* scan range and MS source conditions via continual infusion of standard solution (as outlined in the Materials and methods). Incorporation of all these modifications yielded an optimised method with sufficient resolution and sensitivity to directly quantify 5-AZA-CdR intracellularly (Figure 1d).

### Establishing the multiparameter AZA-MS method

**Figure 2:**
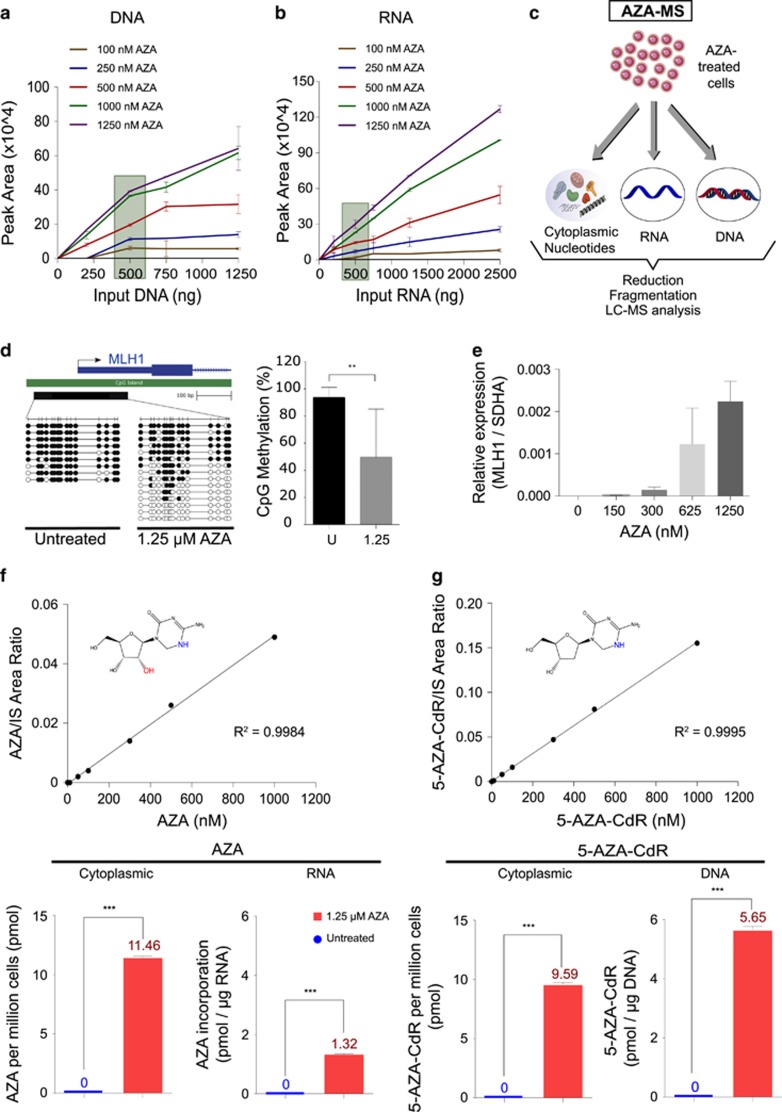
Applying the AZA-MS method to study an *in vitro* AZA-treated cell line. (**a**) Two-factor experiment to determine minimum input amount of DNA required to reliably detect DNA-5-AZA-CdR. Signal intensities of DNA-5-AZA-CdR measured from different input quantities of DNA (0–1250 ng, *x* axis) extracted from RKO cells treated with different concentrations of AZA (100−1250 nM) for 3 consecutive days. A quantity of 500 ng (highlighted with a shaded box) was determined as the minimum amount of DNA to reproducibly detect good signal across all the tested AZA treatment dosages. Points represent mean of three independent experiments, and whiskers represent s.d. (**b**) Experiment to determine minimum input amount of RNA required to reliably detect RNA-incorporated AZA. Signal intensities of RNA-incorporated AZA measured from different input quantities of RNA (0–1250 ng, *x* axis) extracted from RKO cells treated with different concentrations of AZA (100−1250 nM) for 3 consecutive days. A quantity of 500 ng (highlighted with a shaded box) was determined as the minimum amount of RNA to reproducibly detect good signal across all the tested AZA treatment dosages. Points represent mean of three independent experiments, and whiskers represent s.d. (**c**) Schematic of the AZA-MS assay, illustrating the separation of the various subcellular components (cytoplasmic nucleotides, RNA and DNA) from the same sample before LC–MS. (**d**) Allelic bisulphite sequencing of the MLH1 locus in RKO cells. Top panel shows the schematic of the gene, with a CpG island (green box) and the region assayed for CpG methylation (black box). Lollipop visualisations of the methylation status in control, DMSO treated cells (‘untreated’, left panel) and RKO cells treated for 3 days 1.25 μM AZA (right panel) are shown. Equivalent bar-graph quantification of the data is presented on the right. Whiskers represent s.d. ** *P-*value <0.05, Student’s *t*-test. (**e**) Quantitative real-time PCR (qRT-PCR) of MLH1 expression levels in RKO cells treated with different doses of AZA (0–1250 nM) for 3 days, showing robust re-expression after treatment with 1250 nM AZA. (**f**) AZA quantification in cytoplasm and RNA from RKO cells, treated either with 1.25 μM of AZA (red graphs) or control (DMSO, blue graphs) for 3 days. The calibration curve used for AZA quantification is shown, along with AZA chemical structure and the *R*^2^ value. Abundance measurements for AZA in the cytoplasm (left graph) and incorporated into RNA (right graph) are shown. Data are from triplicate experiments, with s.d. depicted by whiskers. ***P-value <0.001, Student’s *t*-test. (**g**) 5-AZA-CdR quantification in cytoplasm and RNA from RKO cells, treated either with 1.25 μM of AZA (red graphs) or control (DMSO, blue graphs) for 3 days. The calibration curve used for 5-AZA-CdR quantification is shown, along with 5-AZA-CdR chemical structure and the *R*^2^ value. Abundance measurements for 5-AZA-CdR in the cytoplasm (left graph) and incorporated into DNA (right graph) are shown. Data are from triplicate experiments, with s.d. depicted by whiskers. ***P-value <0.001, Student’s *t*-test.

As a first step towards eventually applying our method in primary samples, we needed to establish a minimum quantity of input DNA in which we would still be able to detect 5-AZA-CdR incorporation. We devised a straightforward two-factorial experiment employing the colorectal cancer cell line, RKO, in which the molecular mechanisms following 5-AZA-CdR treatment have been well characterised.^43^ We treated RKO cells with a wide range of AZA concentrations (100−1250 nM) that we envisaged would flank the range of reported *in vivo* dosages.^44^ The cells were quickly washed with buffer containing the cytidine deaminase inhibitor tetrahydrouridine^45, 46^ to dampen any deamination of AZA during the subsequent processing steps. DNA was subsequently isolated from these cells and different input concentrations of DNA (100−1250 ng) were reduced and fragmented as per the method we had established. Across the entire range of AZA treatment dosages, we could reliably detect DNA-incorporated 5-AZA-CdR from a minimum of 500 ng of input DNA (Figure 2a).

We next sought to further expand the capability of the method to also simultaneously detect AZA in RNA, as well as to quantify unincorporated, cytoplasmic AZA and 5-AZA-CdR. Mutations or altered expression of the enzymes involved in AZA metabolism have been attributed as causes of AZA and 5-AZA-CdR resistance in cell lines and patients.^18, 47^ Additionally, drug resistance could also potentially arise because of reduced cellular abundance because of decreased influx. From RKO cells treated across a range of relevant AZA concentrations (100−1250 nM), we isolated intact total RNA and reduced and fragmented as established before and assessed signals for RNA-incorporated AZA across a range of different input amounts of RNA (100−1250 ng, Figure 2b). As with DNA, we established that a minimum input amount of 500 ng of RNA yielded reliably quantifiable AZA signals across the entire range of AZA treatment doses tested (Figure 2b). We also established that the lower limit of quantification for the assay was 10 nM for both 5-AZA-CdR and AZA. Finally, to quantify unincorporated free AZA and 5-AZA-CdR in the cytoplasm, we made a final modification to our method by incorporating a methanol-based extraction step to isolate cytoplasmic nucleotides.^31^ In this final, refined method (henceforth termed ‘AZA-MS’), a fraction of AZA-treated cells is set aside for extracting unincorporated nucleotides from the cytoplasm, whereas DNA and RNA are simultaneously extracted from the remaining cells (Figure 2c). This enables the parallel measurement of multiple parameters within the same sample, including: (1) in DNA: 5-AZA-CdR, methyldeoxycytidine and deoxycytidine (representative chromatograms in Supplementary Figures 3a and b); (2) in RNA: AZA, methylcytidine and cytidine (representative chromatograms in Supplementary Figures 3a and b); and (3) in the cytoplasm: levels of unincorporated 5-AZA-CdR, AZA, methyldeoxycytidine, deoxycytidine, methylcytidine and cytidine.

### Applying AZA-MS to survey intracellular dynamics of AZA treatment *in vitro*

To survey the intracellular dynamics of AZA therapy comprehensively using AZA-MS, we treated RKO cells with 1.25 μM AZA, a dose that we established was sufficient to demethylate DNA (Figure 2d) and reinduce expression of the MLH1 tumour suppressor gene (Figure 2e). AZA- or DMSO-treated cells were harvested and DNA, RNA and cytoplasmic extracts were prepared and analysed by the AZA-MS method. By utilising eight-point standard curves for each analyte, QC samples corresponding to low, medium or high quantities of incorporated AZA/5-AZA-CdR, and by spiking samples with defined quantities of isotopically labelled internal reference standards, we can accurately quantify intracellular AZA and 5-AZA-CdR in different subcellular fractions. The AZA-MS quantifications had robust reproducibility, as evidenced by the high accuracy and precision in inter- and intra-assay runs (Table 3).

Free, unincorporated AZA is detected in the cytoplasm of treated cells at 11.46±0.11 pmol AZA/million cells whereas there was no signal in DMSO control cells (Figure 2f). RNA- AZA was also detected in treated cells (1.32±0.03 pmol of AZA per 1 μg of RNA) with no signal in the DMSO control (Figure 2f and Supplementary Figure 4b). Free 5-AZA-CdR was detected in the cytoplasmic extracts (9.59±0.16 pmol 5-AZA-CdR per 1 million cells) as well as DNA-5-AZA-CdR (5.65±0.12 pmol of 5-AZA-CdR per 1 μg of DNA), whereas no signal was observed in control cells (Figure 2g and Supplementary Figure 4c).

We also explored the relationship between 5-AZA-CdR or AZA levels and DNA or RNA methylation respectively using AZA-MS. RKO cells were treated with a range of AZA concentrations (0−1250 nM) for 3 days and analysed by AZA-MS. The amount of DNA-incorporated 5-AZA-CdR increased linearly with increasing AZA doses (*R*^2^=0.9973, Supplementary Figure 5a), whereas DNA methylation levels decreased linearly and inversely (*R*^2^=0.9483, Supplementary Figure 5b). Our data therefore fit neatly with the well-established DNA demethylating role of AZA. Unexpectedly however, a similar trend was not observed in RNA. AZA incorporation increased proportionally with increasing AZA treatment dosage (*R*^2^=0.9896, Supplementary Figure 5c). Global RNA methylation remained unchanged (Supplementary Figure 5d).

### AZA-MS reveals differences in the intracellular dynamics of AZA during *in vivo* therapy

**Figure 3:**
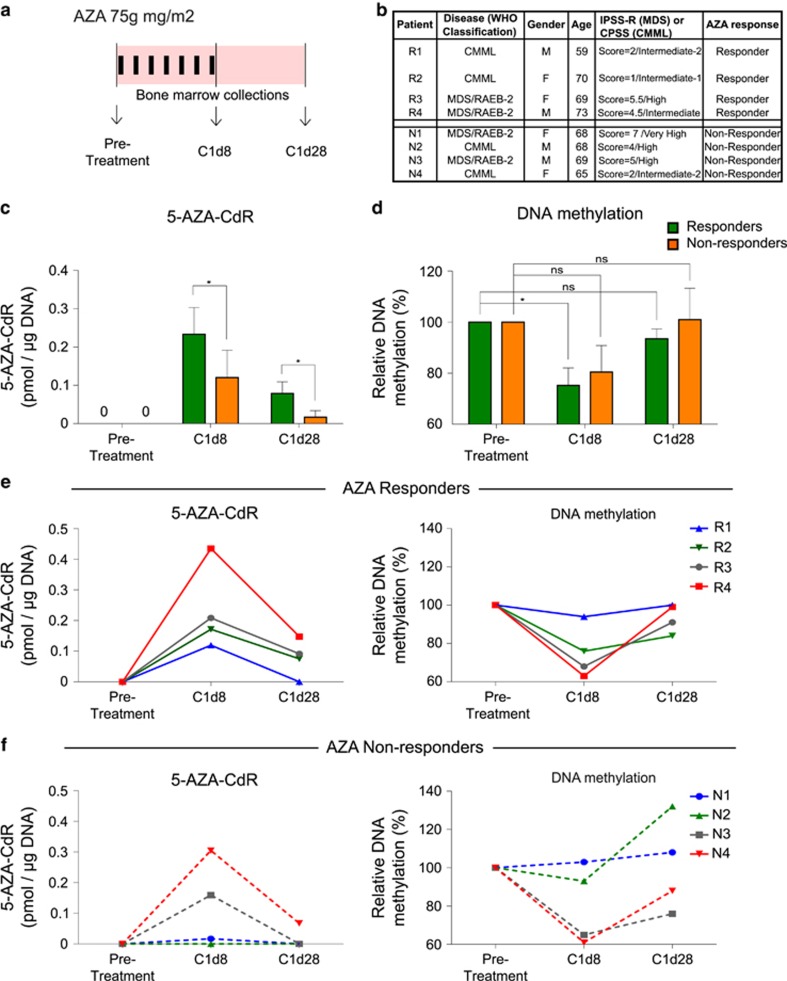
Studying *in vivo* intracellular AZA dynamics using AZA-MS. (**a**) Schematic showing the standard cycle of 7 consecutive days of AZA treatment (black vertical bars) for patients. The three longitudinal time points for collection of bone marrow samples from each patient are also depicted below. Pretreatment, cycle 1 day 8 (C1d8) and cycle 1 day 28 (C1d28). (**b**) Table of patient characteristics. WHO classification,^34^ IPSS-R^35^ and CPSS^36^ scoring and AZA response^37^ were based on published guidelines. (**c**) Bar graphs depicting the mean 5-AZA-CdR abundance in DNA of bone marrow CD34− cells at each of the three time points in AZA responders (*n*=4, green) and nonresponders (*n*=4, orange) are shown, with whiskers showing s.d. *P-value <0.05, Student’s *t*-test. (**d**) Bar graphs representing the mean cytosine methylation levels in DNA of bone marrow CD34− cells at each of the three time points, in AZA responders (*n*=4, green) and nonresponders (*n*=4, orange), with whiskers showing s.d. *P-value <0.05, Student’s *t*-test. (**e**) 5-AZA-CdR abundance (left panel) and DNA methylation levels (right panel) in bone marrow CD34− cells of each of the four AZA responders (R1–R4) shown longitudinally over the course of AZA treatment. (**f**) 5-AZA-CdR abundance (left panel) and DNA methylation levels (right panel) of bone marrow CD34− cells in each of the four AZA nonresponders (N1–N4) shown longitudinally over the course of AZA treatment.

We applied AZA-MS to study AZA intracellular dynamics *in vivo*, using primary samples from MDS or CMML patients undergoing AZA therapy. Bone marrow samples were obtained from eight patients (MDS, *n*=4; CMML, *n*=4) who had all received at least 6 cycles of standard AZA therapy. From each patient, three longitudinal bone marrow samples had been collected over the course of treatment: immediately before the start of treatment, that is, pretreatment; after 7 consecutive days of the first cycle of AZA therapy, C1d8; and at the end of the first cycle of treatment (C1d28), at a period after 20 days off the drug (Figure 3a). Four patients (R1−R4) were assessed to have been AZA complete responders by the IWG (International Working Group) criteria, whereas the other four (N1−N4) were nonresponders (Figure 3b). In preliminary AZA-MS experiments with archived bone marrow samples from AZA-treated samples, we determined that 5 μg of DNA and RNA per experiment reliably yielded DNA-5-AZA-CdR- and RNA-AZA incorporation signals above the limits of detection (Supplementary Figure 5e). Although this quantity of nucleic acid theoretically equates to ∼1 × 10^6^ cells, we determined that a starting quantity of ∼2 × 10^6^ cells was sufficient in practice (Supplementary Figure 5e). It has previously been reported that genome-wide DNA methylation profiles of bone marrow CD34+ and CD34− cells are very similar in MDS.^12^ Consistent with this, we determined that the total DNA methylation levels by AZA-MS were similar in an MDS patient (mean in CD34+ cells=4.8% mean in CD34− cells=5.1% *P*-value=0.39, Supplementary Figure 5f). As sufficient quantities of bone marrow CD34+ cells were not available for all samples, we performed the subsequent AZA-MS analyses in CD34− cells of all patients.

In all eight patients, DNA-5-AZA-CdR could be observed at C1d8, though it was significantly more in AZA responders compared with nonresponders (responders, mean=0.23 pmol of 5-AZA-CdR per μg of DNA; nonresponders, mean=0.12 pmol of 5-AZA-CdR per μg of DNA; *P-*value=0.03, Figure 3c). DNA-5-AZA-CdR level was also correlated with DNA demethylation, with increased demethylation observed in AZA responders (mean=75.3% of pretreatment levels, Figure 3d) compared with nonresponders (mean=80.5% of pretreatment levels, Figure 3d). Although 5-AZA-CdR levels in DNA dropped by C1d28, residual amounts were still detectable in the cells of all patients (responders, mean=0.079 pmol of 5-AZA-CdR per μg of DNA; nonresponders, mean=0.0169 pmol of 5-AZA-CdR per μg of DNA, Figure 3c). DNA methylation, conversely, bounced back to almost pretreatment levels in all patients at C1d28 (Figure 3d).

The patterns of interplay between DNA-5-AZA-CdR and DNA demethylation were even clearer when analysing individual patients. The levels of demethylation at C1d8 were greatest among individuals who had the highest levels of DNA-5-AZA-CdR, both in responders (R4>R3>R2>R1, Figure 3e) and nonresponders (N4>N3>N1 and N2, Figure 3f). Furthermore, two patterns were observed in the nonresponders: in nonresponders N1 and N2, there were very low levels of DNA-5-AZA-CdR at C1d8 (0.01 and 0 pmol of 5-AZA-CdR per μg of DNA respectively, Figure 3f). Consequently, there was no demethylation in these patients (Figure 3f). In the remaining two nonresponders N3 and N4, however, DNA-5-AZA-CdR was much higher (0.16 and 0.3 pmol of 5-AZA-CdR per μg of DNA respectively, Figure 3f) and so was DNA demethylation, dropping to ~60% of pretreatment levels (Figure 3f).

### Low 5-AZA-CdR incorporation into DNA is not a result of impaired intracellular AZA metabolism

**Figure 4:**
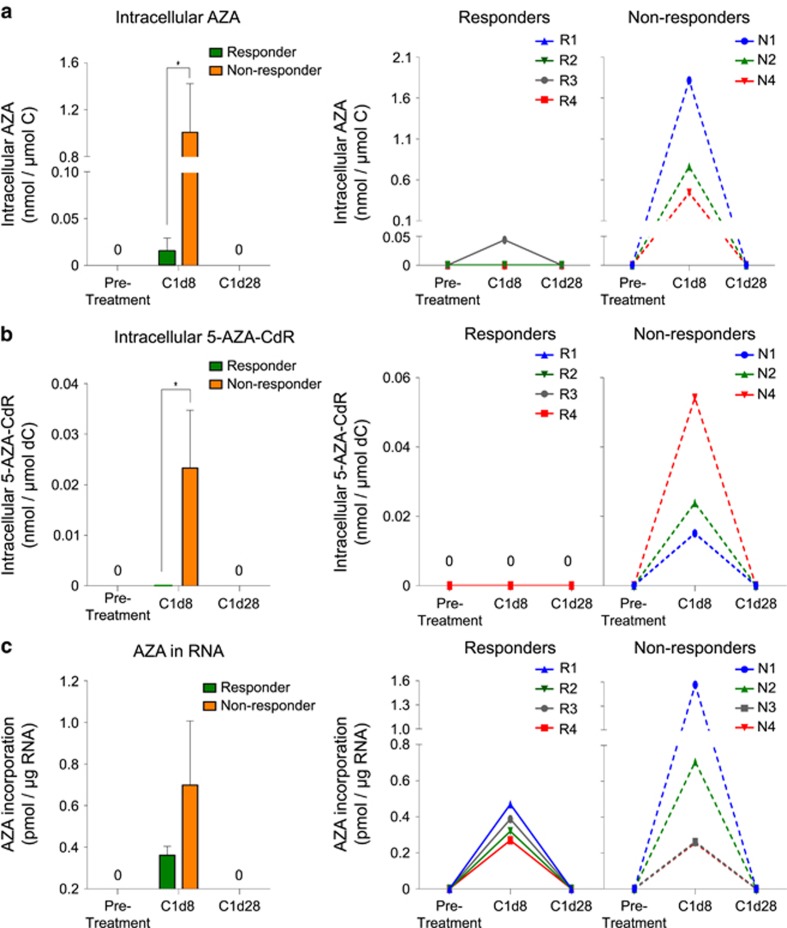
Measuring cytoplasmic and RNA incorporation rates of AZA and 5-AZA-CdR following *in vivo* therapy. (**a**) Bar graphs representing mean abundance measurements of unincorporated AZA in the cytoplasm of bone marrow CD34− cells of AZA responders (*n*=4, green) and nonresponders (*n*=3, orange) at different time points during AZA therapy. Whiskers represent s.d., and *P-value <0.05, Student’s *t*-test. Individual patient measurements are shown on the right. (**b**) Mean abundance measurements of unincorporated 5-AZA-CdR in the cytoplasm of bone marrow CD34− cells of AZA responders (*n*=4, green) and nonresponders (*n*=3, orange). Whiskers represent s.d., and *P-value <0.05, Student’s *t*-test. Individual patient measurements are shown on the right. (**c**) Mean abundance measurements of RNA-incorporated AZA in bone marrow CD34− cells of AZA responders (*n*=4, green) and nonresponders (*n*=4, orange). Whiskers represent s.d., and *P-value <0.05, Student’s *t*-test. Individual patient measurements are shown on the right.

The low DNA level of 5-AZA-CdR observed in the AZA nonresponders could arise because of low intracellular accumulation of AZA, either because of ineffective drug import or elevated drug efflux, or because of low conversion of AZA diphosphate into 5-AZA-CdR diphosphate intracellularly. To investigate these possibilities, we compared the intracellular concentrations of AZA and 5-AZA-CdR in the bone marrow CD34− cells of responders and nonresponders. The levels of intracellular AZA at C1d8 were almost 70-fold higher in nonresponders compared with responders (responders, mean=0.0146 nmol of AZA per μmol cytidine; nonresponders, mean=1.0 nmol of AZA per μmol cytidine, Figure 4a). In fact, nonresponders N1 and N2, who had almost no DNA-5-AZA-CdR, had the highest quantities of intracellular AZA at C1d8 (Figure 4a). Intracellular concentrations of 5-AZA-CdR were also much higher at C1d8 in AZA nonresponders, whereas it was undetectable in the responders (responders, mean=0 nmol of 5-AZA-CdR per μmol deoxcytidine; nonresponders, mean=0.023 nmol of 5-AZA-CdR per μmol deoxcytidine, Figure 4b). Interestingly, in the nonresponders, the patients with the highest levels of free 5-AZA-CdR (N4>N2>N1, Figure 4b) had the lowest levels of free AZA (N1>N2>N4, Figure 4a). The increased amount of unincorporated AZA in nonresponders was also reflected by greater amount of RNA-AZA (responders, mean=0.36 pmol of AZA per μg RNA; nonresponders, mean=0.70 pmol of AZA per μg RNA, Figure 4c). The nonresponders with the highest amounts of free AZA, N1 and N2, also had the highest amounts of RNA- AZA (1.57 and 0.71 pmol of AZA per μg RNA respectively, Figure 4c). This is likely a result of the shift in azacitidine/cytidine nucleotide ratios in the cytoplasm of nonresponders, enabling a greater likelihood for incorporation into transcripts in AZA non-responders.

We have recently shown that the differential expression of a set of 20 cell cycle genes in bone marrow CD34+ cells at pretreatment could efficiently dichotomise AZA responders and nonresponders, on the basis that AZA nonresponders have higher fraction of quiescent progenitor cells.^19^ To explore the link between cell cycle quiescence and DNA-5-AZA-CdR, we determined the expression of this set of cell cycle genes in pretreatment CD34+ cells of all eight patients by quantitative real-time PCR. Consistent with our earlier report,^19^ we found that expression of these genes was higher in the AZA responders (R1−R4) compared with the nonresponders (N1−N4, Supplementary Figure 5g). Furthermore, there was good correspondence between the expression of these genes at pretreatment and the subsequent levels of DNA-5-AZA-CdR measured by AZA-MS at C1d8. Responder R4, who had the highest levels of DNA-5-AZA-CdR (Figure 3e), also had the highest relative expression of the cell cycle genes (Supplementary Figure 5g). Conversely, the two nonresponders with the lowest amounts of DNA-5-AZA-CdR (N1 and N2, Figure 3f) had the lowest relative expression of these genes (Supplementary Figure 5g). Nonresponders N3 and N4, in whom DNA-5-AZA-CdR could be detected by AZA-MS (Figure 3f), had intermediate expression levels of these genes (Supplementary Figure 5g).

## Discussion

Utilising ultra-high mass accuracy LC–MS and developing an optimised method permitting high sensitivity of detection of AZA and 5-AZA-CdR, we have established the AZA-MS method. The high-confidence direct measurements we can make have enabled us to trace the intracellular fate of AZA and quantify its abundance in different subcellular compartments (DNA, RNA and the cytoplasm), while also simultaneously measuring its biological impact through the measurement of DNA and RNA methylation. Previous methods have focussed on smaller set of parameters,^27, 30, 31, 32, 33, 44, 48, 49^ limiting a fuller understanding of the effects of AZA on the cell. The closest efforts to measuring as many multiple parameters as we have done with AZA-MS have either been (1) through scintillation counting, *ex vivo* treating cells with tritiated AZA, followed by measuring radiolabelled 5-AZA-CdR in DNA and intracellularly;^30^ or (2) using mass spectrometry, but only to measure 5-AZA-CdR in DNA and corresponding DNA methylation following decitabine treatment *in vitro* (of cell lines) or *in vivo* in mice.^48^ The former method is inapplicable for studying unlabelled AZA given therapeutically and it is unclear what artefactual effects *ex vivo* treatment of cells would have. The latter method did not quantify unincorporated 5-AZA-CdR in the cytoplasm and its direct suitability to examine human samples remains unclear. The comprehensive simultaneous measurements of multiple parameters by AZA-MS yield the fullest insights into the intracellular dynamics of AZA treatment. Futures modifications of the method, such as by altering the enzymatic mix used for digesting nucleic acids, together with appropriate internal standards, might even permit a comprehensive kinetic analysis of the intracellular metabolism of AZA or 5-AZA-CdR to be studied systematically. The AZA-MS method could also be adapted to measure other nucleic acid modifications of clinical and biological importance, including DNA hydroxymethylation or RNA adenine methylation, as well as to study the intracellular pharmacology of other nucleotide analogues used as therapeutic agents.

Applying AZA-MS to clinically annotated patient samples, we observed that the biggest difference between AZA responders and nonresponders was the uniformly higher levels of DNA-5-AZA-CdR in AZA responders. The magnitude of DNA-5-AZA-CdR also neatly correlated with DNA demethylation. However, we observed two patterns in AZA nonresponders: some AZA nonresponders showed minimal DNA-5-AZA-CdR and minimal DNA demethylation. In these patients, we could detect AZA and DNA-5-AZA-CdR intracellularly, as well as RNA-AZA, suggesting that neither cellular uptake nor intracellular metabolism could be potential reasons to explain the low DNA-5-AZA-CdR in these patients. Our examination of the cell cycle status suggests that in these patients, an increased proportion of the bone marrow cells might be quiescent and not undergoing DNA replication,^19, 20^ resulting in low levels of DNA-5-AZA-CdR.

However, other AZA nonresponders showed DNA-5-AZA-CdR and DNA demethylation at levels that were comparable to levels in AZA responders. It has recently been reported that 5-AZA-CdR treatment of cancer cell lines induces demethylation and transcription of endogenous retroviral elements, leading to an interferon response in cells.^16, 17^ It is possible that AZA nonresponders with DNA-5-AZA-CdR fail to respond to AZA therapy because of a failure to induce an interferon response that is necessary for clinical response. Alternatively, these patients could have increased tolerance to, or defective, immune cell-mediated clearance of dysplastic cells.^50^

The objective of this study was to develop a method that enabled us to make direct measurements in patient samples to better understand clinical response to AZA. Using this method, we have observed for the first time that AZA refractoriness may not simply be because of failure of AZA uptake in cells and incorporation in DNA but is more complex. Use of this assay in conjunction with future prospective clinical trials that involve AZA or decitabine therapy will help tease out the biological complexities that underlie drug refractoriness and help personalise future treatment options for MDS/CMML.

## Figures and Tables

**Table 1:** UHPLC gradient conditions

*Time (min)*	*A (%)*^a^	*B (%)*^b^
0	100	0
2.5	100	0
3	0	100
3.5	0	100
4	100	0
8	100	0

Abbreviation: UHPLC, ultra-high-performance liquid chromatography.

a A: 0.1% formic acid in Milli-Q water.

b B: 0.1% formic acid in acetonitrile.

**Table 2:** Analytes of interest and their corresponding monoisotopic masses

*Analyte*	*m/z*	*Analytes*	*m/z*
Dihydro-AZA	247.1037	Deoxycytidine (dC)	228.09788
Dihydro-AZA-^15^N_4_	251.09242	5-Methyldeoxycytidine (mdC)	242.11353
Dihydro-5-AZA-CdR	231.10878	Cytidine (C)	244.0928
Dihydro-5-AZA-CdR-^15^N_4_	235.09747	5-Methylcytidine (mC)	258.10845

Abbreviation: AZA, 5-azacytidine.

**Table 3:** Quality control measurements

*Analyte*			*Lower limit of quantification*	*Low QC*	*Medium QC*	*High QC*
			*10 nM*	*30 nM*	*300 nM*	*1000 nM*
Dihydro-5-AZA-CdR		Average±s.d.	11.3±4.33	31±2.83	299±1.78	998±2.28
	*Intra*-day	Accuracy (%)	96.7–103.4	99.9–101.6	99.8–100.2	99.76–100.1
		Precision (%)	0–8.7	2.1–2.2	1.1–1.8	0.30–0.37
	Inter-day	Accuracy (%)	102.5	101.8	99.28	100.36
		Precision (%)	7.1	1.75	1.26	0.85
Dihydro-5-AZA			*10 nM*	*50 nM*	*300 nM*	*1000 nM*
		Average±s.d.	9.3±4.2	51±3.73	299±2.49	997±3.21
	Intra-day	Accuracy (%)	93.3–102.4	102.7–105.1	99.45–99.98	100.1–100.8
		Precision (%)	6.11–9.25	4.8–9.3	0.53–1.1	0.74–5.82
	Inter-day	Accuracy (%)	95.42	99.69	99.42	100.23
		Precision (%)	6.91	8.7	3.59	0.49

Abbreviations: AZA, 5-azacytidine; QC, quality control.
