# Supplementary material for: AZA-MS: a novel multiparameter mass spectrometry method to determine the intracellular dynamics of azacitidine therapy in vivo
Source: Leukemia. 2018 Jan 16;32(4):900–10. doi: 10.1038/leu.2017.340 (PMC5886051; doi:10.1038/leu.2017.340)

SUPPLEMENTARY FIGURE LEGENDS

Supplementary Figure 1. Ineffective resolution of 5-AZA-CdR from dC by TQMS. (A.) Representative selected reaction monitoring chromatograms from liquid chromatography-triple quadrupole mass spectrometry (TQMS) for buffer spiked with 1 µM 5-AZA-CdR only (left) or 1 µM dC only (right). The m/z for dC (top chromatograms, left and right panels) and 5-AZA-CdR (bottom chromatograms, left and right panels) are indicated, as are the retention times (RT) for the molecules. (B) Representative mass spectrum of TQMS at RT of 1.34 min indicating that TQMS cannot separate signals from 5-AZA-CdR (black peak) and the naturally occurring isotopes of dC (red peak) due to poor mass resolution.

Supplementary Figure 2. Method optimisation to improve sensitivity of 5-AZA-CdR detection by mass spectrometry. (A) Scatter plot of quantification of 5-AZA-CdR standards of different concentrations (0.5-1000 nM 5-AZA-CdR), showing the effect of 37°C incubation on 5-AZA-CdR signal intensity. Two identical sets of standards were analysed, one prepared fresh without any incubation prior to MS ("No incubation", blue line), and the other incubated for six hours at 37°C prior to MS ("With incubation", red line). Data from three independent experiments is shown, with whiskers corresponding to standard deviation. (B) Gel electrophoresis image showing the fragmentation efficiency for different combinations of DNA amount and incubation time. Four incubation times were examined (0, 1, 2, and 6 hours) for each of the three DNA masses (1, 2, and 5 µg). (C) Scatter plot showing the improvement in 5-AZA-CdR LC-MS signal due to of $NaBH_4$ reduction. Two identical sets of standards were analysed, one without any reduction ("No reduction", blue line), and the other with reduction ("With reduction", red line). Data from three independent experiments is shown, with whiskers corresponding to standard deviation. (D.) Representative chromatograms from an AZA-MS

run on an Orbitrap, illustrating complete reduction of 1000 nM of 5-AZA-CdR (middle panel) to dihydro5-AZA-CdR (bottom panel). A reference chromatogram (top panel) of a fully converted isotopically labelleled internal standard (iSTD, dihydro-5-AZA-CdR-$^{15}N_4$) is also provided. The noise level (NL) is also provided for reference. (E.) Representative chromatograms illustrating complete reduction of 1000 nM of 5-AZA (middle panel) to dihydro-AZA (bottom panel). A reference chromatogram of a fully converted isotopically labelled internal standard (iSTD, dihydro-AZA-$^{15}N_4$) is also provided. (F) Scatter plot showing the improvement in 5-AZA-CdR LC-MS signal due to omission of ammonium formate. Two identical sets of standards were analysed, one with ammonium formate (blue line), and the other without (red line). Data from three independent experiments is shown, with whiskers corresponding to standard deviation.

Supplementary Figure 3. Representative AZA-MS chromatograms. (A.) Representative chromatograms of the analytes dihydro-AZA and dihydro-5-AZA-CdR, along with internal standards. (B.) Representative chromatograms of the deoxyribonucleoside analytes, dC and methyl dC, as well as the equivalent ribonucleoside analytes C and methylC.

Supplementary Figure 4. Quality control measurements in AZA-MS. (A.) Representative chromatograms of quality control samples, showing analyte specific peaks. The top panel of chromatograms illustrates genomic DNA from untreated cells, spiked with 1000 nM 5-AZA-CdR, along with relevant internal standard. The bottom panel of chromatograms illustrates RNA from untreated cells, spiked with 1000 nM 5-AZA, along with relevant internal standard. (B.) Chromatograms for the relevant deoxyribonucleoside analytes (5-AZA-CdR, dC and methyl dC) from genomic DNA of untreated cells, illustrating specificity of detection. (C.)

Chromatograms for the ribonucleoside analytes (5-AZA, C and methylC) in RNA of untreated cells, illustrating specificity of detection.

Supplementary Figure 5. Studying the dynamics between 5-AZA-CdR or AZA incorporation and methylation. (A) Scatter plots showing a linear positive correlation between increasing AZA treatment dosage (x-axis) and increased 5-AZA-CdR incorporation into DNA (y-axis) in RKO cells treated for three days. Points represent mean from triplicate experiments, with whiskers representing standard deviation. (B) Linear negative trend between increasing AZA treatment dosage (x-axis) and decreased DNA cytosine methylation (y-axis) in RKO cells treated for three days. Points represent mean from triplicate experiments, with whiskers representing standard deviation. (C) Scatter plots showing a linear positive correlation between increasing AZA treatment dosage (x-axis) and increased AZA incorporation into RNA (y-axis) in RKO cells treated for three days. Points represent mean from triplicate experiments, with whiskers representing standard deviation. (D) Scatter plots showing no correlation between increasing AZA treatment dosage (x-axis) and RNA cytosine methylation (y-axis) in AZA treated RKO cells. Points represent mean from triplicate experiments, with whiskers representing standard deviation. (E) Recommended starting material for AZA-MS. (F) DNA cytosine methylation (%) in CD34+ and CD34- BM fractions from a MDS patient on AZA (day 8). (G) Hierarchical clustering of patients based on normalised qPCR expression levels of 20 cell cycle genes. N= non-responder, R= responder.

Supplementary Figure 1

A.

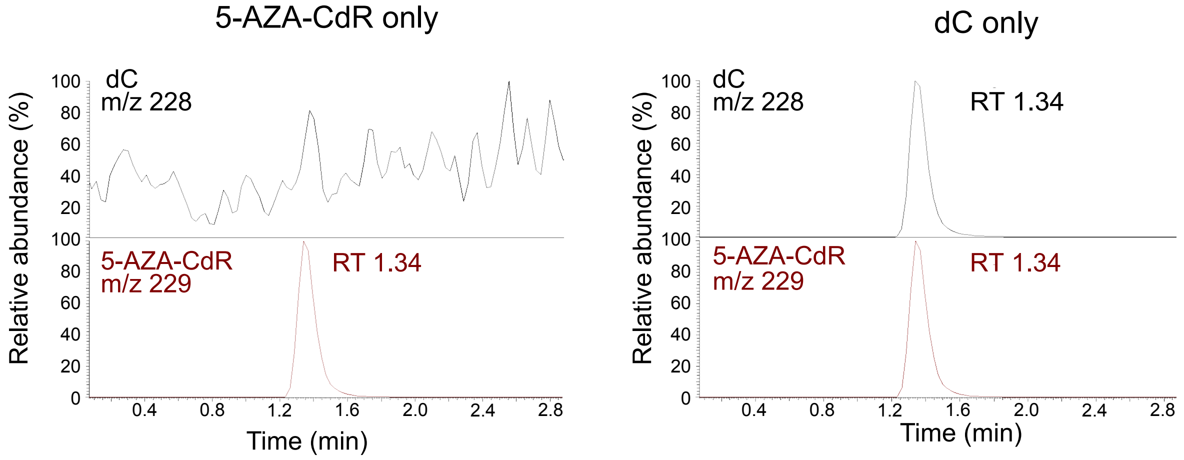

B.

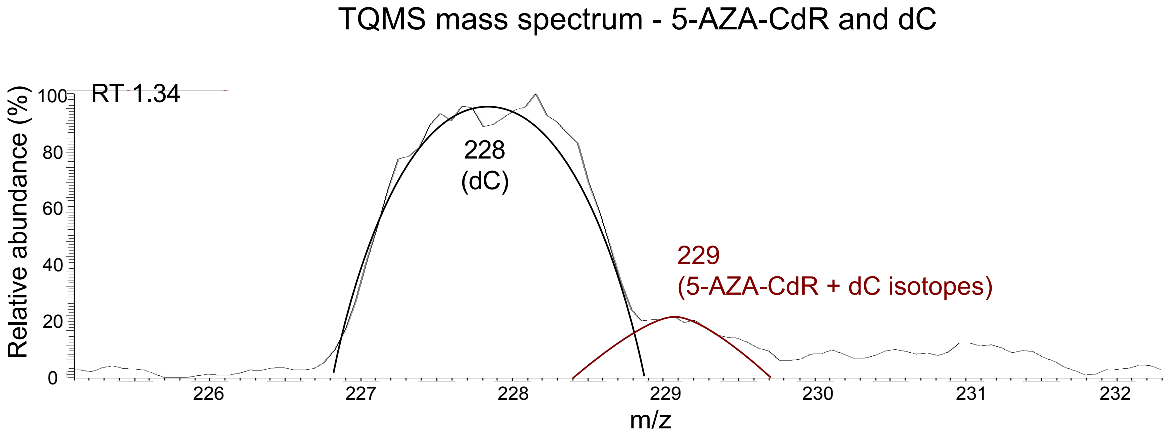

Supplementary Figure 2

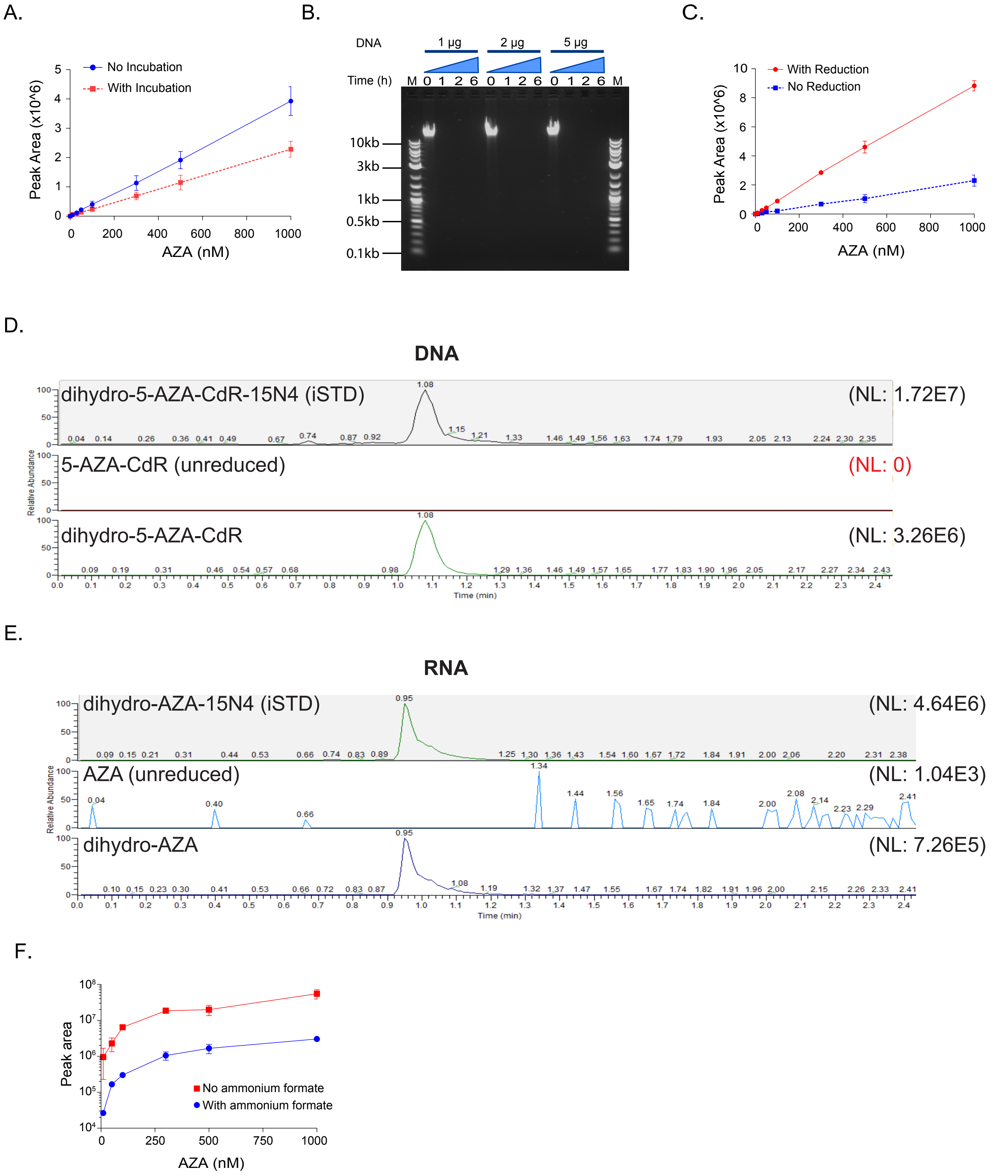

A.

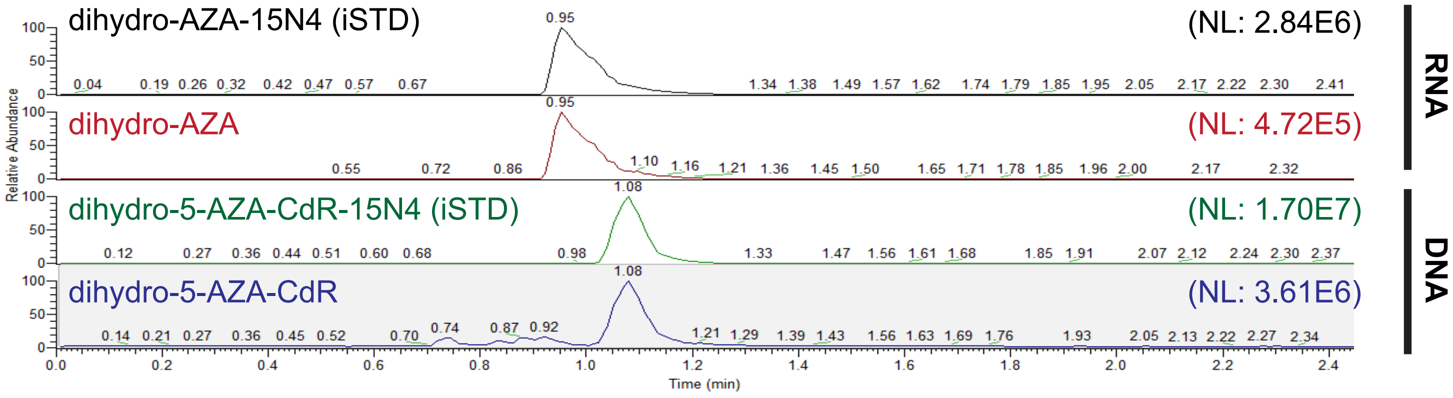
dihydro-AZA-15N4 (iSTD)
(NL: 2.84E6)
dihydro-AZA
(NL: 4.72E5)
dihydro-5-AZA-CdR-15N4 (iSTD)
(NL: 1.70E7)
dihydro-5-AZA-CdR
(NL: 3.61E6)
RNA
DNA
Relative Abundance
Time (min)

B.

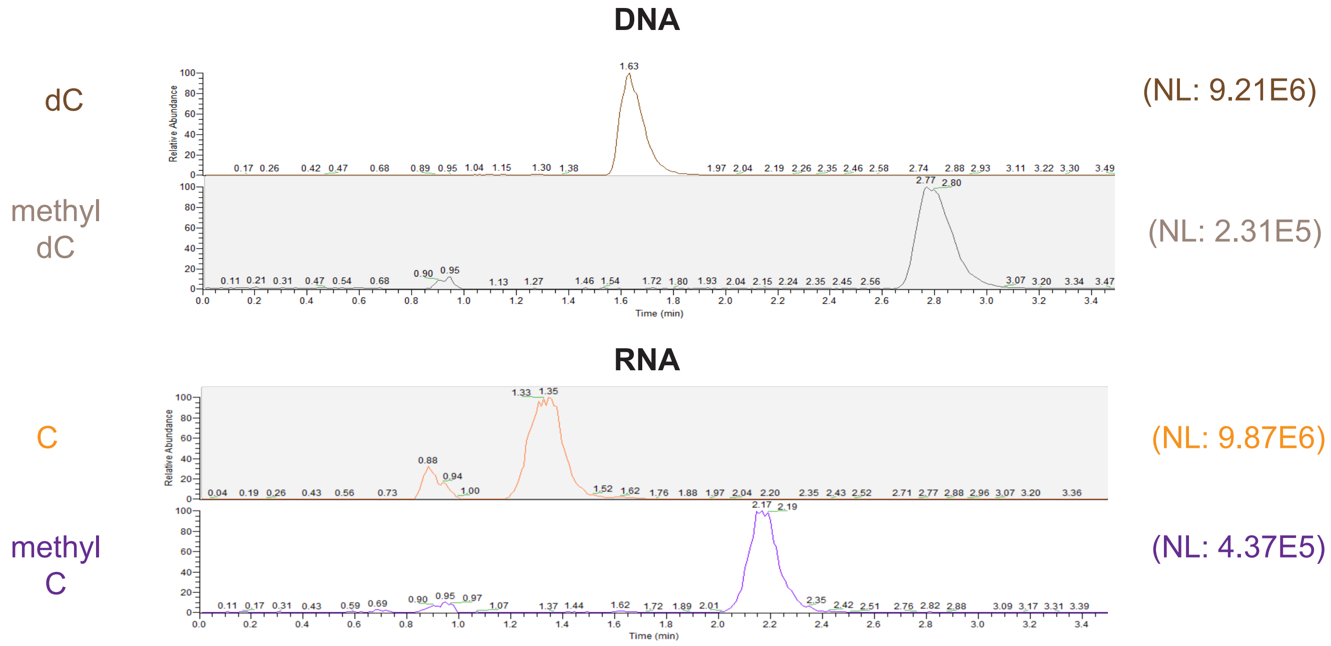
DNA
dC
(NL: 9.21E6)
methyl dC
(NL: 2.31E5)
RNA
C
(NL: 9.87E6)
methyl C
(NL: 4.37E5)
Relative Abundance
Time (min)

A.

Spike-in controls

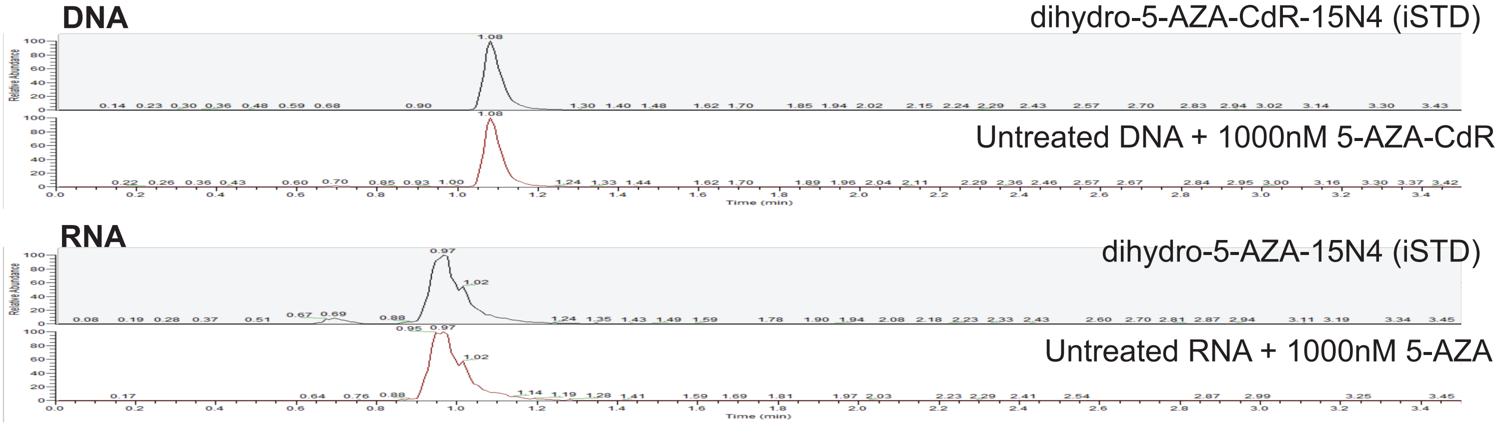

B.

RNA from untreated cells

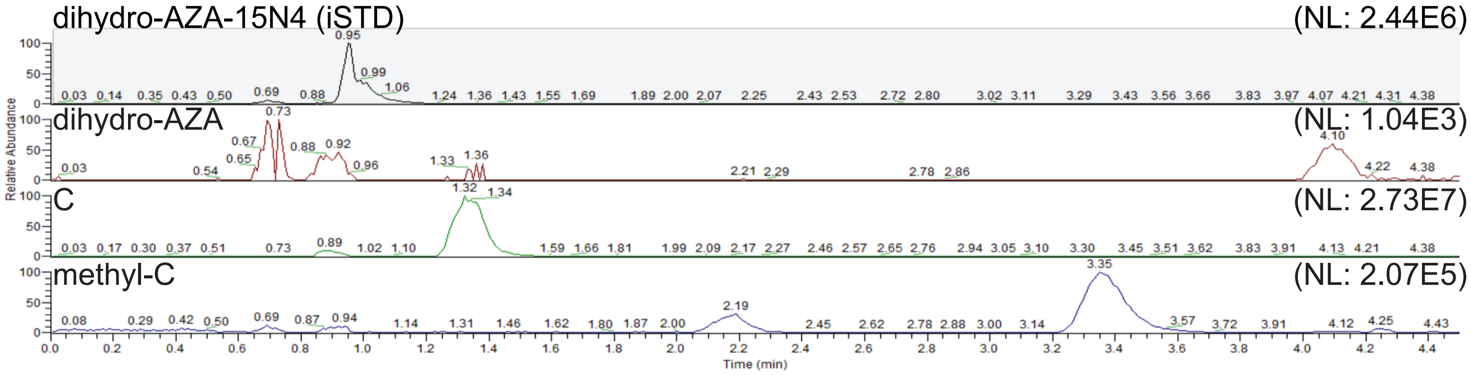

C.

DNA from untreated cells

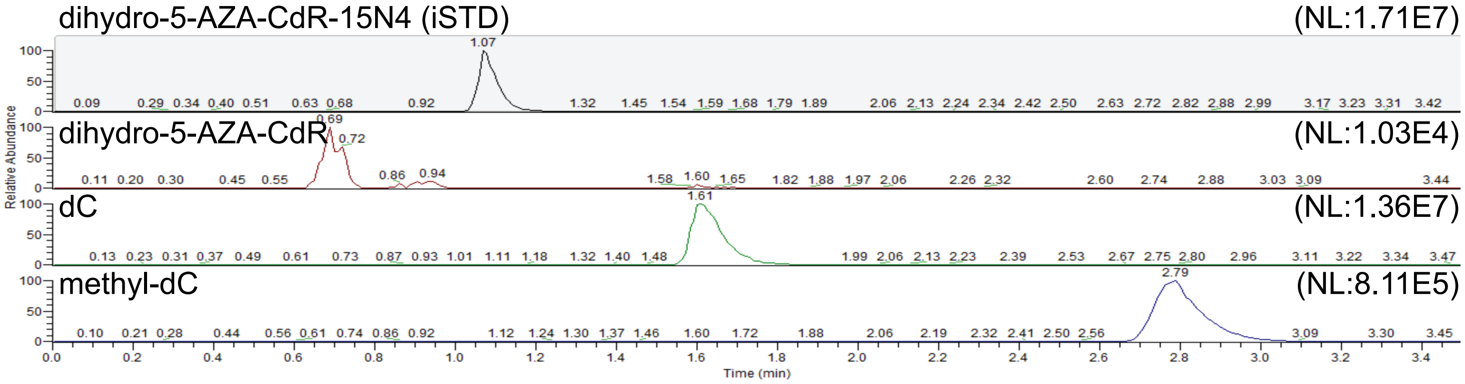

A.

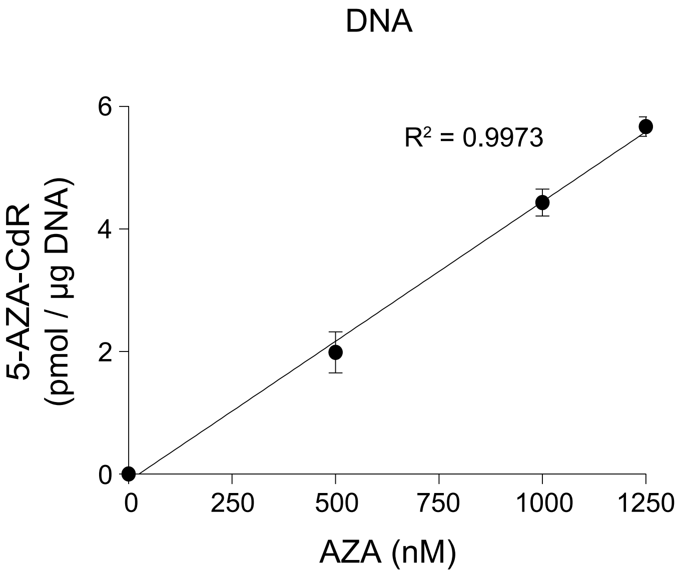

B.

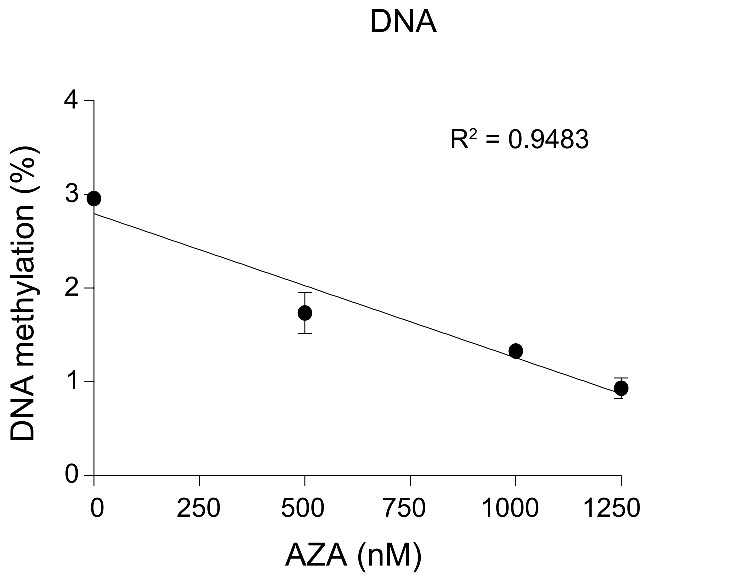

C.

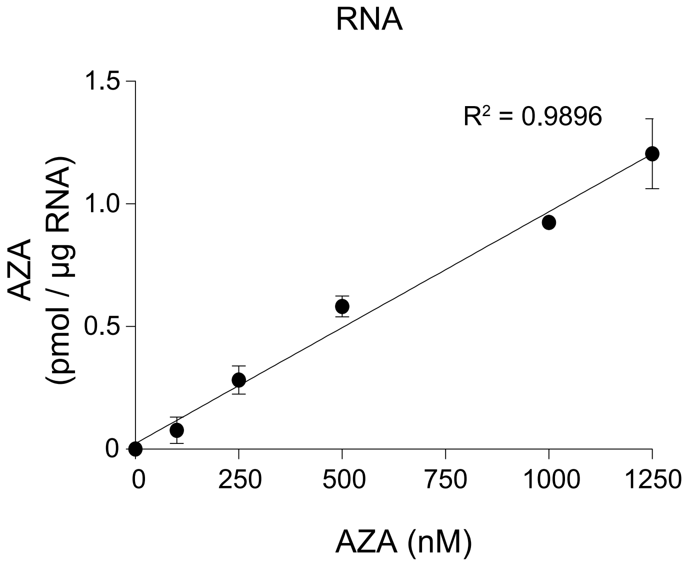

D.

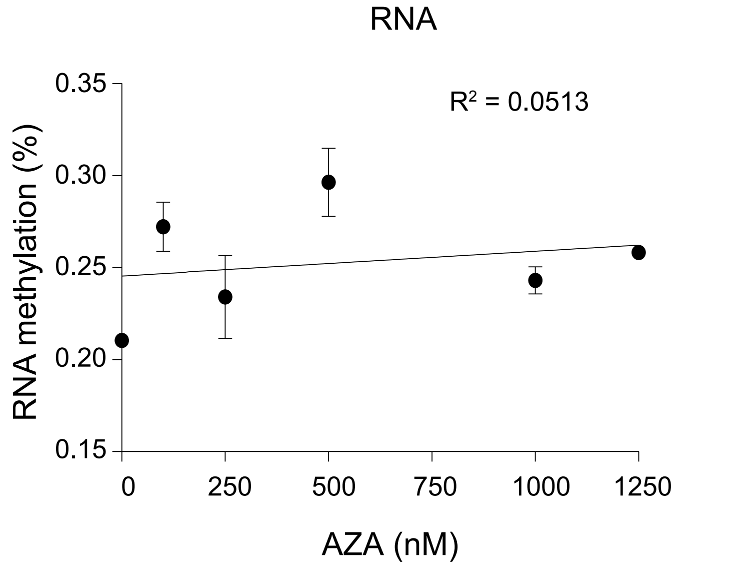

E.

	Sub-cellular Fraction	Recommended quantity of analyte	Theoretical quantity required (x10^6 cells)	Practical quantity recommended (x10^6 cells)
**Cell lines**	DNA<br>RNA<br>Cytoplasm	} 500 ng<br>> 50nM	0.1	~0.5<br>~0.5
**Bone Marrow Samples**	DNA<br>RNA<br>Cytoplasm	} 5 µg<br>> 50nM	1	~2<br>~2

F.

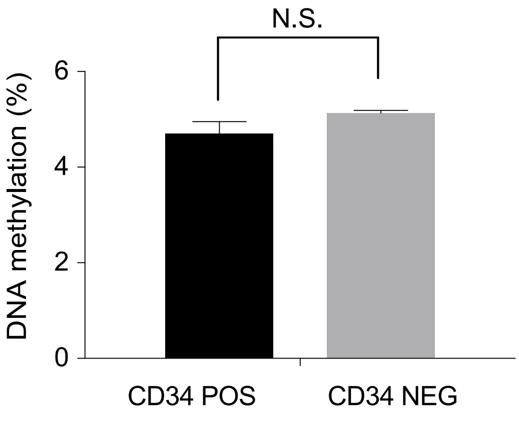

G.

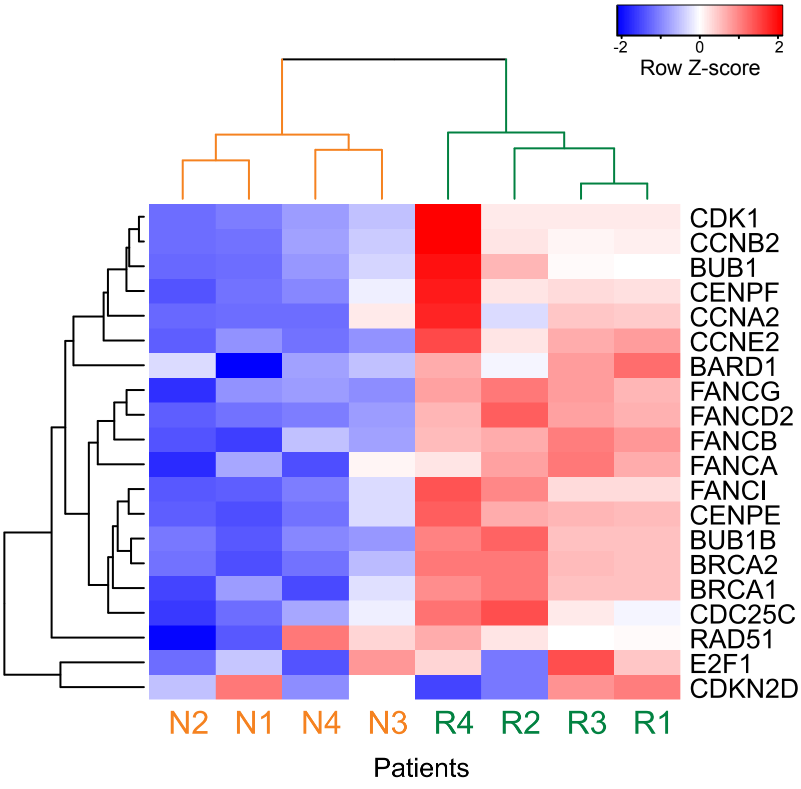

Supplement: Supplementary Material [file leu2017340x1.pdf]
